# Genome-Wide Identification and Characterization of NAC Family in *Hibiscus hamabo* Sieb. et Zucc. under Various Abiotic Stresses

**DOI:** 10.3390/ijms23063055

**Published:** 2022-03-11

**Authors:** Zhiquan Wang, Longjie Ni, Dina Liu, Zekai Fu, Jianfeng Hua, Zhiguo Lu, Liangqin Liu, Yunlong Yin, Huogen Li, Chunsun Gu

**Affiliations:** 1 Institute of Botany, Jiangsu Province and Chinese Academy of Sciences, Nanjing 210014, China; wangzhiquan@cnbg.net (Z.W.); jfhua@cnbg.net (J.H.); lzgjs@cnbg.net (Z.L.); liuliangqin@cnbg.net (L.L.); ylyin@cnbg.net (Y.Y.); 2 College of Forest Sciences, Nanjing Forestry University, Nanjing 210037, China; longjieni@njfu.edu.cn (L.N.); cc0212@njfu.edu.cn (D.L.); fzk@njfu.edu.cn (Z.F.); hgli@njfu.edu.cn (H.L.); 3 Jiangsu Key Laboratory for the Research and Utilization of Plant Resources, Jiangsu Provincial Platform for Conservation and Utilization of Agricultural Germplasm, Nanjing 210014, China

**Keywords:** NAC family, transcription factors, abiotic stress, semi-mangrove plant, *Hibiscus hamabo*

## Abstract

NAC transcription factor is one of the largest plant gene families, participating in the regulation of plant biological and abiotic stresses. In this study, 182 NAC proteins (HhNACs) were identified based on genomic datasets of *Hibiscus hamabo* Sieb. et Zucc (*H. hamabo*). These proteins were divided into 19 subfamilies based on their phylogenetic relationship, motif pattern, and gene structure analysis. Expression analysis with RNA-seq revealed that most *HhNACs* were expressed in response to drought and salt stress. Research of quantitative real-time PCR analysis of nine selected *HhNACs* supported the transcriptome data’s dependability and suggested that *HhNAC54* was significantly upregulated under multiple abiotic stresses. Overexpression of *HhNAC54* in *Arabidopsis thaliana* (*A. thaliana*) significantly increased its tolerance to salt. This study provides a basis for a comprehensive analysis of NAC transcription factor and insight into the abiotic stress response mechanism in *H. hamabo*.

## 1. Introduction

NAC transcription factor is one of the largest plant gene families. The most notable structural feature of NAC transcription factor is the presence of a highly conserved NAC domain (about 150–160 amino acids) at the N-terminus of the protein, while the C-terminus is a modified transcriptional regulatory region (TAR) [1]. It participates in the regulation of plant stress responses and life processes such as plant leaf senescence, flower formation, fruit maturation and coloring, seed development, and root development. For example, tomato *SlNAC6* is involved in drought stress response and the reproductive process [2]. Pepper *CaNAC035* is a positive regulator of abiotic stress tolerance [3], and *CaNAC064* modulates plant tolerance to cold stress [4]. Rice *ONAC127* and *ONAC129* are involved in the grain filling process [5], and *OsNAC2* participates in ABA-induced leaf senescence [6]. Wheat *TaNAC30* negatively regulates resistance to stripe rust [7], and *TaNAC2-5A* controls the nitrate response and increases wheat yield [8]. Because of the development of high-throughput sequencing technology, an increasing number of plants have completed whole-genome sequencing. Therefore, the identification research of NAC has entered an explosive period. Research on the identification and expression of related gene families has been carried out in *Triticum aestivum* [9], *Fagopyrum tataricum* [10], *Betula pendula* [11], *Prunus mume* [12], *Panicum virgatum* [13], and other species.

Studying the responses of plants to abiotic stress is helpful in better protecting and utilizing plants. *Hibiscus hamabo* Sieb. et Zucc. (*H. hamabo*) is a semi-mangrove plant belonging to *Hibiscus Maliace*. It is extremely resistant to salt and alkalis, as well as drought and barren environments, and it has developed roots. Moreover, it is a pioneer tree species with excellent wind resistance and embankment protection in coastal areas. It is a woody plant that integrates medicinal, edible, health care, ornamental, greening, water and soil protection, and fibrous raw material values [14]. Our team has completed the whole genome sequencing of *H. hamabo* (not yet published). On this basis, we have carried out a genome-wide identification of the NAC transcription factor gene family, and we have verified the function of the *HhNAC54* gene. This study has certain significance for the analysis of the molecular response mechanism of *H. hamabo* under abiotic stress, and it can also provide an excellent gene reserve for the molecular selection of salt-tolerant plants.

## 2. Results

### 2.1. Identification and Analysis of HhNAC Genes in H. hamabo

Based on the genomic information of *H. hamabo*, 185 putative *NAC* genes were obtained with HMM. Three genes without the NAC and NAM domain were eliminated according to the results from the SMART and Pfam databases, leaving 182 *HhNAC* genes (Table S1). The lengths of protein sequences, chromosome locations, start and stop sites, protein molecular weights, and isoelectric points (pI) were examined (Table S2). The results showed that 182 genes were randomly distributed on 45 chromosomes, except chromosome 35, and they were named *HhNAC1*–*HhNAC182* according to their positions on the chromosomes. The protein lengths were between 203 (HhNAC79) and 799 (HhNAC170) amino acids, and the protein molecular weights were between 23,021.5 (HhNAC79) and 88,094.4 Da (HhNAC170).

**Figure 1:**
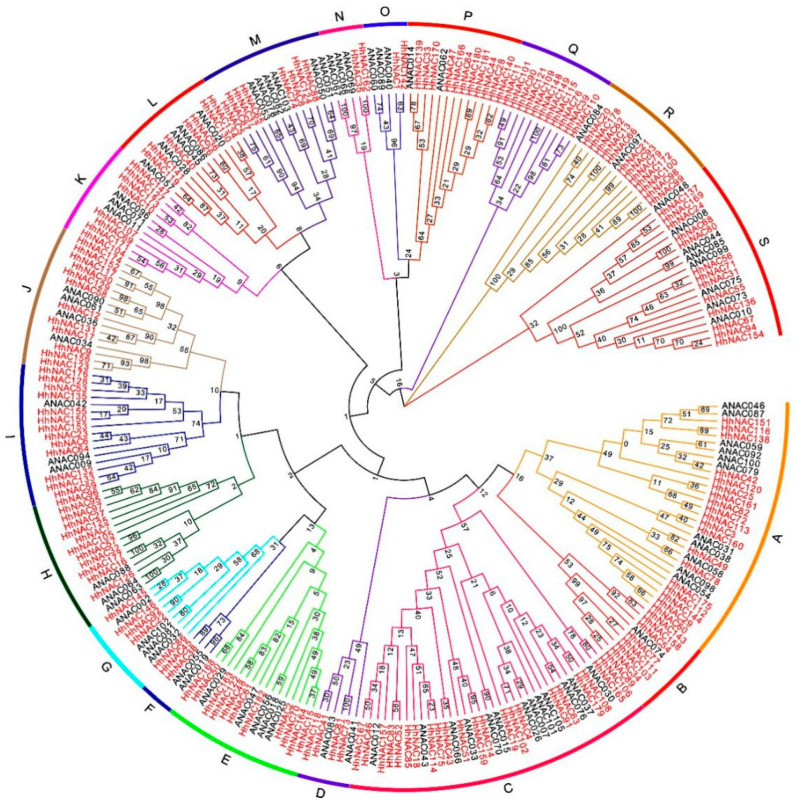
Phylogenetic tree of NAC proteins in *A. thaliana* and *H. hamabo*.

NAC domains were found to cover a range of 40–50 amino acids with a high similarity compared to HhNAC protein domains. To further study the evolutionary relationships, we compared 182 HhNAC and AtNAC protein sequences containing an NAC domain (Figure 1). The 182 HhNACs could be classified into 19 subfamilies (A-S), of which the C subfamily contained the most members (22), whereas the O, N and F subfamilies contained only two members each. In addition, HhNAC subfamilies were expanded or contracted to different degrees compared with *A. thaliana*. For example, 0 members belonged to the Q subfamily in *A. thaliana*, but the number increased to 11 in *H. hamabo*. This situation also appeared in B, R, C, H, I, J, E, and P subfamilies and others, whereas in the O and N subfamilies, the number of HhNACs decreased significantly.

**Figure 2:**
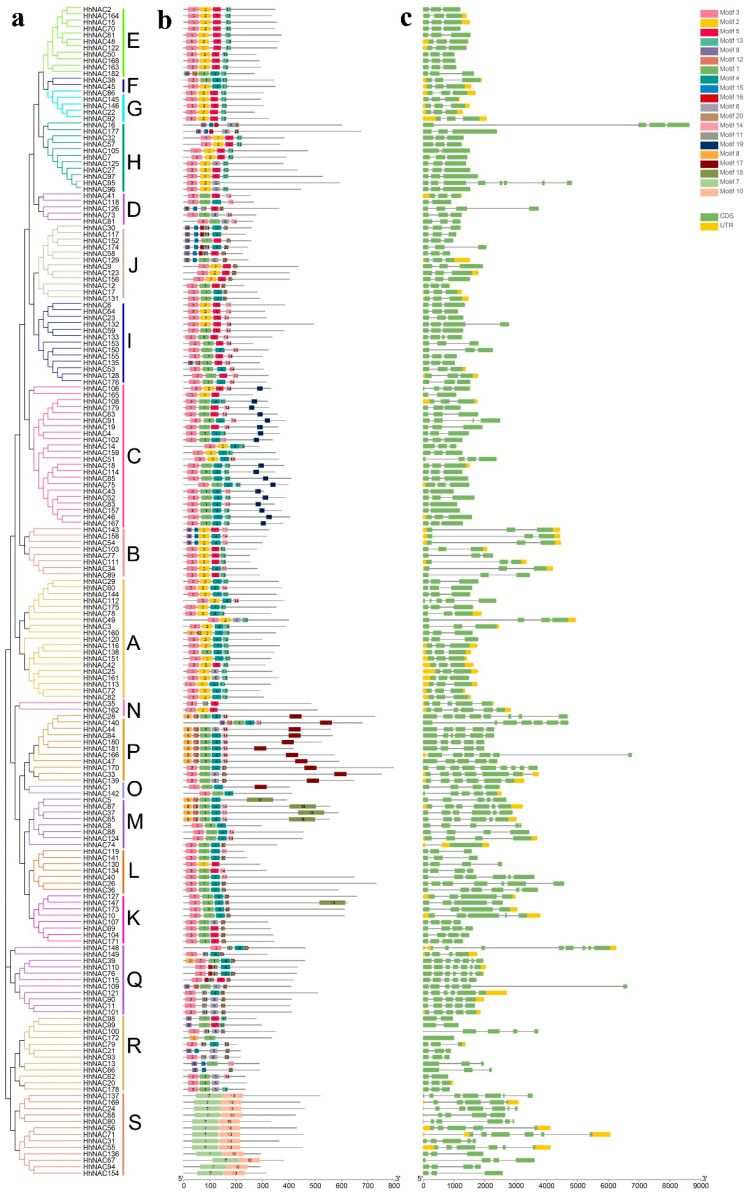
Map of the gene structure distribution and conserved motif patterns in *H. hamabo* NAC proteins. (**a**) Phylogenetic tree of HhNAC (A-S indicated 19 subfamilies). (**b**) Schematic diagram of HhNAC motif distribution. There were 20 types of motif in total, and different motifs are represented by different colors. (**c**) Schematic diagram of the *HhNAC* gene structure.

The motif compositions of 182 HhNAC proteins were analyzed using an online MEME tool to better understand their conserved structures and evolutionary relationships and ultimately revealed 20 distinct motifs (Figure 2). Corresponding with the result of the phylogenetic analysis, the components of the same subfamily were formed by similar motifs. Except in the widely distributed Motifs 2, 3, 5, and 13, HhNAC members in the same subfamily have similar motifs, such as subfamilies A, B, C, and S, suggesting that the function of HhNAC proteins in the same subfamily may be similar. In addition, some motifs only appear in a specific subfamily, such as Motif 7 and Motif 10, which only appear in subfamily S, and Motif 17, which only appears in subfamily P. These motifs may be important in specific subfamilies, which are worthy of further study. A few HhNAC proteins with different motifs were classified in the same branch, indicating that these HhNACs may have undergone functional differentiation in the evolution process. The structural study of HhNAC is further performed in this research (Figure 2). A typical structure containing two exons existed in most *HhNAC* members (104 *HhNAC*s), and the intron number of the remaining *HhNAC*s ranged from 0 to 17, indicating the functional differentiation of *HhNAC* family members. Similarly conserved motifs and gene structures in HhNACs of the same subfamilies further confirmed the reliability of evolutionary relationships.

**Figure 3:**
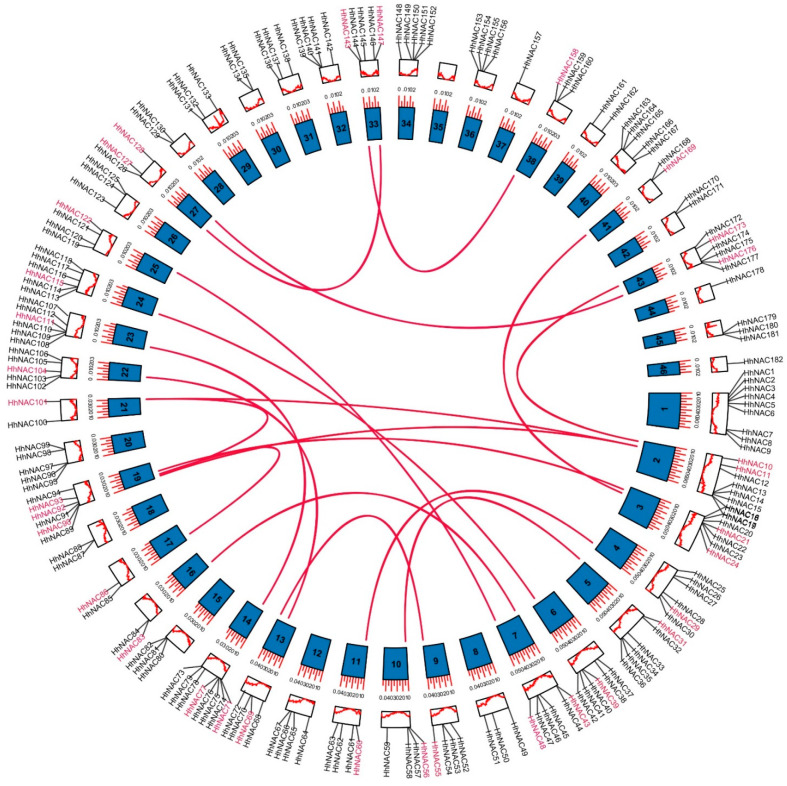
Chromosome locations and collinear relationships of *HhNAC* genes. The inner circle represents different chromosomes. The red lines in the inner circle indicate genes exhibiting collinearity.

According to the annotation information, 182 *HhNAC* genes were annotated on 45 chromosomes (Figure 3). *HhNAC* genes are distributed on 45 chromosomes, except chromosome 35, among which chromosome 1 contained the highest number of genes (9 *HhNAC*s), whereas some chromosomes only contained one *HhNAC* gene, such as Chromosomes 37, 44, and 46. In addition, a positive correlation was not found between the number of genes and the length of the chromosome. In order to explore the evolutionary process of *HhNAC* genes, intragenomic synteny analysis was conducted, and a total of 18 pairs of *HhNAC* genes were found to have a collinear relationship (Figure 3). The substitution rate (Ka/Ks) of the 18 pairs of gene synonymous (Ks) and non-synonymous (Ka) mutations was calculated, which ranged from 0.16 to 0.47, with an average of 0.28, suggesting that purifying the selection was key to the evolution of the *HhNAC* family.

**Figure 4:**
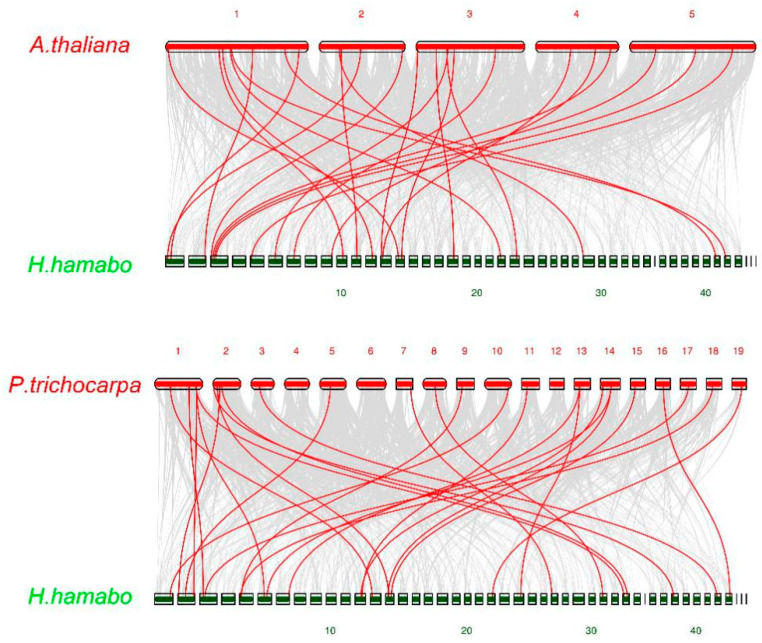
Synteny analysis maps of *H. hamabo* with *A. thaliana* and *P. trichocarpa*. Gray lines indicate the collinear blocks within *H. hamabo* and other plant genomes, whereas the red lines highlight the syntenic *NAC* gene pairs.

In order to understand the phylogenetic mechanism of *HhNAC*s, we performed synteny analysis of *HhNAC*s with *NAC* genes in *A. thaliana* and *Populus trichocarpa*. There were 24 and 26 *HhNAC* genes in a syntenic relationship with *A. thaliana* and *P. trichocarpa*, respectively (Figure 4). These genes may be important in evolution of the *NAC* gene family. In addition, some syntenic gene pairs were anchored to highly conserved syntenic blocks, spanning more than 100 genes, indicating that there may be an evolutionary relationship between *H. hamabo* and two other plants.

### 2.2. Expression Patterns of HhNAC Genes in Different Abiotic Stresses

**Figure 5:**
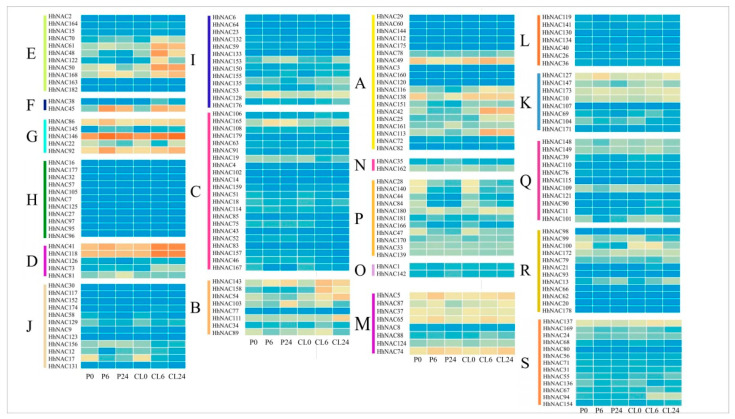
Heat maps of the expression profiles of all *HhNAC* genes under drought and salt stress (A-S indicated 19 subfamilies). The expression abundance (in log10-based FPKM) of each transcript is represented by the color: orange, higher expression; blue, lower expression. P0 and CL0 = 0 h of drought and salt stress, P6 and CL6 = 6 h of drought and salt stress, and P24 and CL24 = 24 h of drought and salt stress.

To further explore the biological function of *HhNAC*s, the expression level of 182 *HhNAC* genes were analyzed under drought and salt stress, which was based on the transcriptomic data (Figure 5). Only a few *HhNAC*s were not expressed in all samples (FPKM = 0), and the expressed genes (FPKM > 0) showed significant differences in expression pattern during different treatments and time points. Some genes exhibited the same pattern of expression under drought and salt stress, as *HhNAC70* and *HhNAC168* of the E subfamily, *HhNAC146* of the G subfamily, and *HhNAC41*, *HhNAC73* and *HhNAC118* of the D subfamily were all upregulated under the two stresses, whereas *HhNAC17* of the J subfamily, *HhNAC44* of the P subfamily, and *HhNAC100* of the R subfamily were downregulated under both stress conditions. Several genes showed opposing expression trends under the two types of stress. *HhNAC103* of subfamily B was induced under PEG treatment and reduced under NaCl treatment. *HhNAC50* and *HhNAC158* of subfamily E were induced under NaCl treatment and reduced under PEG treatment. The results suggest that they may participate in the stress response process in different ways.

**Figure 6:**
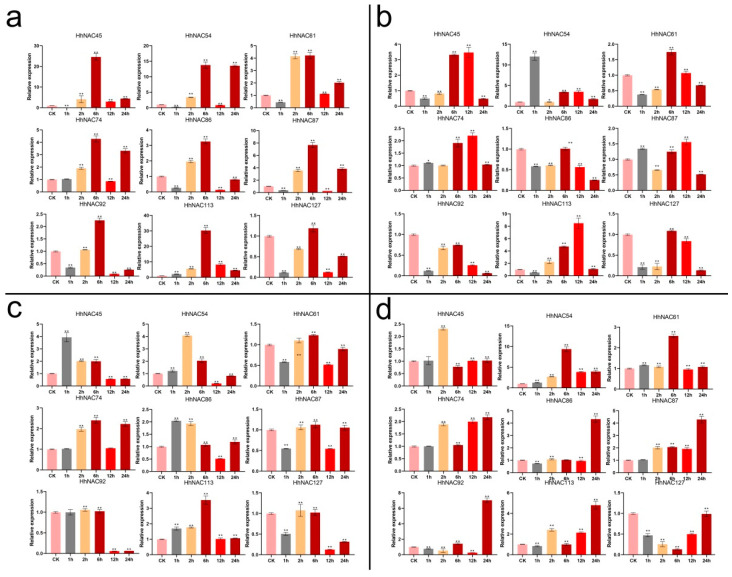
*HhNAC* genes were analyzed by qRT-PCR. The significance analysis was carried out using Student’s *t*-test (* *p* < 0.05, ** *p* < 0.01).

Plant responses to drought and salt stress are also influenced by ABA and MeJA signals. Nine genes with the largest fold changes under the PEG and NaCl treatments were selected for qRT-PCR analysis, so that *H. hamabo* responses to salt, drought stresses, and ABA and MeJA treatments could be studied at six time points (Figure 6). The expression trends of these nine genes at the same time point in all samples showed consistency with the transcriptome, supporting the transcriptome result’s dependability. Meanwhile, some genes showed different expression trends in the early stage of the stress response. For example, *HhNAC45* and *HhNAC61* had higher expression levels at 6 h under salt stress, which began to decrease after 6 h, thus indicating that they may play different roles in the early and late stages of the stress response. Some *HhNAC* genes could be significantly induced or suppressed by the four treatments, such as *HhNAC54* and *HhNAC61*. Furthermore, several genes exhibited opposing expression trends when responding to distinct environments. *HhNAC127*, for example, was upregulated under PEG and NaCl stress, but downregulated under ABA and MeJA treatments. These findings suggested that the *HhNAC* family participated in multiple stress responses and had diverse regulatory mechanisms.

### 2.3. HhNAC54 Overexpression Increases Transgenic A. thaliana Salt Tolerance

**Figure 7:**
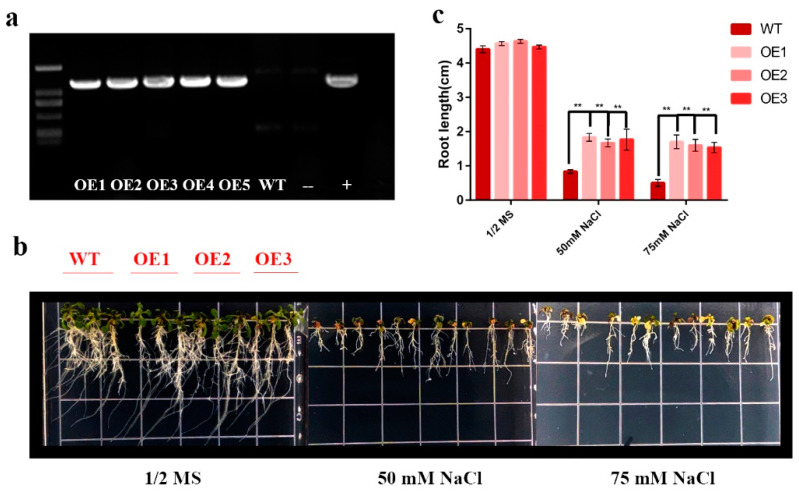
Salt tolerance assay of *HhNAC54*-overexpressed *A. thaliana* plants. (**a**) Agarose gel electrophoresis profile for the expression of *HhNAC54*. (**b**) Comparison between root lengths of WT and transgenic *A. thaliana* under different concentrations of NaCl. (**c**) Statistic histogram of root length in WT and transgenic *A. thaliana* under different concentrations of NaCl (** *p* <0.01).

Because of the expression pattern under the four treatments, the *HhNAC54*-overexpressing plants were reinforced with *A. thaliana* to study whether the expression of *HhNAC54* could improve the salt tolerance (Figure 7). Five T_3_ transgenic plants were obtained through resistance screening. The PCR was set for detecting and determining the expression of *HhNAC54* in T_3_ plants. Three independent transgenic lines were selected for subsequent studies (OE1-3). Transgenic plants and wild types (WT) were grown on a 1/2 MS medium with progressively elevated salt concentrations. There was no obvious difference in the root length of transgenic plants and WT planted on normal 1/2 MS medium after 10 d. Root growths of transgenic plants were less inhibited by high salinity than those of wild type, showing that resistance to salt stress in the transgenic *A. thaliana* was increased, and *HhNAC54* had a function in improving salt tolerance.

## 3. Discussion

The NAC transcription factor mainly exists in terrestrial plants [15]. NAC family was identified in *H. hamabo* for the first time in this study, and the member quantity (182) was more than that in *A. thaliana* (117), rice (151) [16], and soybean (152) [17]. The phenomenon of multiple members may be caused by the extensive duplication and diversification of the genome during evolution [18]. The gene family can be augmented in a variety of ways, including genome-wide replication, tandem replication, fragment replication, and others [18].

HhNAC members can be divided into 19 subfamilies, and almost all subfamilies contained both HhNAC and AtNAC proteins. Differences in the number of gene subfamily members between *H. hamabo* and *A. thaliana* may be due to genes that are copied or lost. Genes clustered in the same subfamily were closely related, which indicates that they might have similar functions. The evolutionary relationship between HhNAC members can also be analyzed by comparing conserved motifs. In this study, the HhNACs in the same subfamily shared common motifs, which indicates that they were highly conserved, further proving the reliability of HhNAC classification.

In recent years, many transcription factors have been identified as enhancing plant adaption to stress [19,20]. A large amount of evidence indicated that the NAC family played a key role in determining plant responses to abiotic stress. For example, the overexpression of *NAC* enhanced rice tolerance to salt and drought stress [21]. The wheat *TaSNAC4* homeologous gene *TaSNAC4-3A* was induced under a series of abiotic stresses, and the overexpression of *TaSNAC4-3A* in *A. thaliana* can endow it with a tolerance to drought stress [22]. The expression trends of *HhNAC* genes were analyzed based on transcriptome data from roots exposed to drought and salt stress. The expression trends of most *HhNAC* genes after drought and salt stress exhibited significantly different levels of expression. Some genes exhibited the same expression trend under the two types of stresses, indicating that they might play similar roles in responding to the two stresses. Some genes showed opposite expression trends, indicating that they might play completely different roles. qRT-PCR analyses in this study were performed to further verify gene expression levels under stresses. Moreover, the additional three time points were set in order to compare the expression patterns in the early and late stages of the response to stresses.

Based on the results of the transcriptome data and qRT-PCR analysis, *HhNAC54* was selected for further functional analysis using transgenic *A. thaliana*. The changes in the root length of the transgenic *A. thaliana* were significantly lower than those of the WT, which indicates that *HhNAC54* had a function in improving salt tolerance, and that the regulation mechanism of *HhNAC54* is worth further studying.

## 4. Materials and Methods

### 4.1. Plants Materials and Treatments

*H. hamabo* was taken from the Institute of Botany, Chinese Academy of Sciences, Jiangsu Province. After 20 days of vernalization, the plump and healthy *H. hamabo* seeds were soaked in concentrated sulfuric acid for 15 min and then washed with tap water repeatedly. The treated seeds then were sown onto plugs, the substrate was composed of peat and vermiculite (1:1) in the pH range of 5–6, and they were cultured in a light incubator (Volume: 1008 L) with a photoperiod of 16/8 h (day (12,000 lux intensity white light LEDs)/night), and a temperature of 24/20 °C under a relative humidity of 65%. The *H. hamabo* seedlings that grew 8~10 true leaves were treated. The seedlings were taken out of the container, cleaned, transferred to a hydroponic culture in 1/2 MS solution for 7 d, and then subjected to abiotic stress treatment in the container with same light intensity, photoperiod, and temperature.

Treatment 1: 400 mM NaCl. Treatment 2: 15% polyethylene glycol (PEG 6000). Treatment 3: 50 μM abscisic acid (ABA). Treatment 4: 1 mM methyl jasmonate (MeJA). Treatments 3 and 4 were spraying seedlings evenly. After the treatment, the leaves were collected at 1 h, 2 h, 6 h, 12 h, and 24 h, frozen quickly in liquid nitrogen, and then stored at −80 °C for later use. Each treatment has three biological replicates.

### 4.2. Identification and Analysis of NAC Genes in H. hamabo

Based on the whole genome data of *H. hamabo* (not yet published), NAM domain (PF02365) and NAC (PF01849) Hidden Markov Model (HMM) profiles from the Pfam database (http://pfam.xfam.org, accessed on 16 March 2021) were used as probes to screen *NAC* genes in *H. hamabo* by HMMER (v. 3.1) software, and the E value was set to E < 10^−3^ [23,24]. ClustalW software (v2.1) was used for performing a multiple sequence alignment with the obtained NAC candidate sequences to remove redundant sequences [25,26]. MEGA7.0 software was used to compare the NAC protein sequences of *H. hamabo* and *A. thaliana* [27,28]. The neighbor-joining method was selected for constructing the phylogenetic tree. The parameter of random sampling repetition times was set to 1000 bootstrap, and the system default values were used for other parameters.

The GSDS website (http://gsds.cbi.pku.edu.cn, accessed on 16 March 2021)) was used to draw a schematic diagram of the *NAC* gene structure [29]. MEME (http://meme-suite.org/, accessed on 16 March 2021) was made to find the conservative motif and draw its protein secondary structure [30]. The parameter is set to 10 motifs. TBtools software (v. 1.089, JAVA, China) was used to draw the gene structure map and for chromosome mapping [31]. MCScanX software was used to analyze collinearity, tandem repeats, and the homologous evolution among different species, and Circos software was used for drawing [32,33]. KaKs_Calculator (v.2.0) software was used to calculate the Ks and Ka/Ks values between gene pairs [34].

### 4.3. Quantitative Real-Time PCR

Quantitative real-time PCR was determined using the mature method [35]. The PCR instrument and reagent were a StepOnePlus real-time PCR system (Applied Biosystems) and a SYBR Green Master Mix (Bimake, Houston, TX, USA), respectively. Total RNA was extracted with Plant Rneasy Mini Kit (Qiagen, Hilden, Germany). The first cDNA strand was synthesized by A PrimeScript^®^ RT kit (TaKaRa, Dalian, China). Genscript online design software (https://www.genscript.com/tools/pcr-primers-designer, accessed on 16 March 2021) was used to design the primer pairs (Table S3). A clustered heat map was drawn using Tbtools software. The reference gene was *Actin* (*HhACT*) [14]. Three independent experiments were conducted. Relative transcript abundances were analyzed using the 2^−ΔΔCT^ method [36].

### 4.4. Identification of Transgenic A. thaliana

*HhNAC* transgenic lines had obtained as described previously [37]. In brief, the *HhNAC* ORF was inserted into the pCAMBIA1305 vector using the ClonExpress II One Step Cloning Kit (Vazyme, Nanjing, China). Then, pCAMBIA1305-*HhNAC* was transformed into *Agrobacterium tumefaciens* GV3101 using the freeze–thaw method [38]. Col-0 of *A. thaliana* was transformed via the floral dip method [38]. Hygromycin screened (150 mg/L) homozygous T_3_ transgenic *A. thaliana* lines were used for functional identification.

### 4.5. Statistical Analysis

Student’s *t*-test was selected for significant difference analysis (* *p* < 0.05 and ** *p* < 0.01). GraphPad Prism (v.8.0) software was used to draw histograms.

## 5. Conclusions

This is the first study to identify the *NAC* family in *H. hamabo* at the genome level. A total of 182 *HhNAC*s were obtained, which were then classified into 19 groups based on phylogenetic relationship, motif pattern, and gene structure analysis. The expression patterns of *HhNAC*s under drought and salt stress were analyzed using RNA-seq and qRT-PCR, and the stress response gene *HhNAC54* was obtained for further functional analysis. Overexpression of *HhNAC54* in *A. thaliana* significantly increased the tolerance to salt. This study provided a basis for a comprehensive analysis of the *NAC* gene family and abiotic stress response mechanism in *H. hamabo*.

## Data Availability

Not applicable.

## Supplementary Materials

The following supporting information can be downloaded at: www.mdpi.com/article/10.3390/ijms23063055/s1.

## References

[1] Olsen A.N., Ernst H.A., Leggio L.L., Skriver K. (2005). NAC transcription factors: Structurally distinct, functionally diverse. Trends Plant Sci..

[2] Jian W., Zheng Y., Yu T., Cao H., Chen Y., Cui Q., Xu C., Li Z. (2021). *SlNAC6*, a NAC transcription factor, is involved in drought stress response and reproductive process in tomato. J. Plant Physiol..

[3] Zhang H., Ma F., Wang X., Liu S., Chen R. (2020). Molecular and functional characterization of *CANAC035*, an NAC transcription factor from pepper (*Capsicum annuum* L.). Front. Plant Sci..

[4] Hou X.M., Zhang H.F., Liu S.Y., Wang X.K., Chen R.G. (2019). The NAC transcription factor *CANAC064* is a regulator of cold stress tolerance in peppers. Plant Sci..

[5] Ren Y., Huang Z., Jiang H., Wang Z., Wu F., Xiong Y., Yao J. (2021). A heat stress responsive NAC transcription factor heterodimer plays key roles in rice grain filling. J. Exp. Bot..

[6] Mao C., Lu S., Lv B., Zhang B., Shen J., He J., Luo L., Xi D., Chen X., Ming F. (2017). A rice NAC transcription factor promotes leaf senescence via ABA biosynthesis. Plant Physiol..

[7] Wang B., Wei J., Song N., Wang N., Zhao J., Kang Z. (2018). A novel wheat NAC transcription factor, *TANAC30*, negatively regulates resistance of wheat to stripe rust. J. Integr. Plant Biol..

[8] He X., Qu B., Li W., Zhao X., Teng W., Ma W., Ren Y., Li B., Li Z., Tong Y. (2015). The nitrate-inducible NAC transcription factor *TANAC2-5A* controls nitrate response and increases wheat yield. Plant Physiol..

[9] Guérin C., Roche J., Allard V., Ravel C., Mouzeyar S., Bouzidi M.F. (2019). Genome-wide analysis, expansion and expression of the NAC family under drought and heat stresses in bread wheat (*T. Aestivum* L.). PLoS ONE.

[10] Liu M., Ma Z., Sun W., Huang L., Wu Q., Tang Z., Bu T., Li C., Chen H. (2019). Genome-wide analysis of the NAC transcription factor family in tartary buckwheat (*Fagopyrum tataricum*). BMC Genom..

[11] Chen S., Lin X., Zhang D., Li Q., Zhao X., Chen S. (2019). Genome-wide analysis of NAC gene family in *Betula pendula*. Forests.

[12] Zhuo X., Zheng T., Zhang Z., Zhang Y., Jiang L., Ahmad S., Sun L., Wang J., Cheng T., Zhang Q. (2018). Genome-wide analysis of the NAC transcription factor gene family reveals differential expression patterns and cold-stress responses in the woody plant *Prunus mume*. Genes (Basel).

[13] Yan H., Zhang A., Ye Y., Xu B., Chen J., He X., Wang C., Zhou S., Zhang X., Peng Y. (2017). Genome-wide survey of switchgrass NACs family provides new insights into motif and structure arrangements and reveals stress-related and tissue-specific nacs. Sci. Rep..

[14] Ni L., Wang Z., Liu L., Guo J., Li H., Gu C. (2019). Selection and verification of candidate reference genes for gene expression by quantitative RT-PCR in *Hibiscus hamabo* sieb. Et zucc. Trees.

[15] Zhu T., Nevo E., Sun D., Peng J. (2012). Phylogenetic analyses unravel the evolutionary history of NAC proteins in plants. Evol. Int. J. Org. Evol..

[16] Ling L., Song L., Wang Y., Guo C. (2017). Genome-wide analysis and expression patterns of the NAC transcription factor family in *Medicago truncatula*. Physiol. Mol. Biol. Plants.

[17] Wang Y., Bai X. (2014). Bioinformatics analysis of NAC gene family in *Glycine max* L.. Soybean Sci..

[18] Song J., Gao Z., Huo X., Sun H., Xu Y., Shi T., Ni Z. (2015). Genome-wide identification of the auxin response factor (ARF) gene family and expression analysis of its role associated with pistil development in Japanese apricot (*Prunus mume* sieb. Et zucc). Acta Physiol. Plant..

[19] Gong S., Ding Y., Hu S., Ding L., Chen Z., Zhu C. (2019). The role of HD-ZIP class|transcription factors in plant response to abiotic stresses. Physiol. Plant..

[20] Sahil, Keshan R., Rather S.A. (2021). Transcription factors involved in plant responses to stress adaptation. Frontiers in Plant-Soil Interaction.

[21] Zheng X., Bo C., Lu G., Han B. (2009). Overexpression of a NAC transcription factor enhances rice drought and salt tolerance. Biochem. Biophys. Res. Commun..

[22] Mei F., Chen B., Li F., Zhang Y., Mao H. (2021). Overexpression of the wheat NAC transcription factor *TASNAC4-3A* gene confers drought tolerance in transgenic *Arabidopsis*. Plant Physiol. Biochem..

[23] Eddy S.R. (2011). Accelerated profile hmm searches. PLoS Comput. Biol..

[24] (2015). HMMER.

[25] Aiyar A. (2000). The use of clustal w and clustal x for multiple sequence alignment. Bioinformatics Methods and Protocols.

[26] (2010). ClustalW.

[27] Kumar S., Stecher G., Tamura K. (2016). Mega7: Molecular evolutionary genetics analysis version 7.0 for bigger datasets. Mol. Biol. Evol..

[28] (2016). MEGA.

[29] Guo A.Y., Zhu Q.H., Chen X., Luo J.C. (2007). Gsds: A gene structure display server. Hereditas.

[30] Bailey T.L., Johnson J., Grant C.E., Noble W.S. (2015). The meme suite. Nucleic Acids Res..

[31] Chen C., Chen H., Zhang Y., Thomas H.R., Frank M.H., He Y., Xia R. (2020). Tbtools: An integrative toolkit developed for interactive analyses of big biological data. Mol. Plant.

[32] Wang Y., Tang H., DeBarry J.D., Tan X., Li J., Wang X., Lee T.H., Jin H., Marler B., Guo H. (2012). *Mcscanx*: A toolkit for detection and evolutionary analysis of gene synteny and collinearity. Nucleic Acids Res..

[33] (2012). MCScanX.

[34] Wang D., Zhang Y., Zhang Z., Zhu J., Yu J. (2010). Kaks_calculator 2.0: A toolkit incorporating gamma-series methods and sliding window strategies. Genom. Proteom. Bioinform..

[35] Ni L., Wang Z., Fu Z., Liu D., Yin Y., Li H., Gu C. (2021). Genome-wide analysis of basic helix-loop-helix family genes and expression analysis in response to drought and salt stresses in *Hibiscus hamabo* sieb. Et zucc. Int. J. Mol. Sci..

[36] Gu C., Xu S., Wang Z., Liu L., Zhang Y., Deng Y., Huang S. (2018). De novo sequencing, assembly, and analysis of *Iris. Lactea* var. Chinensis roots’ transcriptome in response to salt stress. Plant Physiol. Biochem..

[37] Gu C., Liu L., Song A., Liu Z., Zhang Y., Huang S. (2018). *Iris lactea* var. Chinensis (fisch.) cysteine-rich gene LLCDT1 enhances cadmium tolerance in yeast cells and *Arabidopsis thaliana*. Ecotoxicol. Environ. Saf..

[38] Gu C., Song A., Zhang X., Wang H., Li T., Chen Y., Jiang J., Chen F., Chen S. (2016). Cloning of chrysanthemum high-affinity nitrate transporter family (*cmnrt2*) and characterization of *cmnrt2.1*. Sci. Rep..

